# Supplementary figures and images for: Rapid On-Site Phenotyping via Field Fluorimeter Detects Differences in Photosynthetic Performance in a Hybrid—Parent Barley Germplasm Set
Source: Sensors (Basel). 2020 Mar 8;20(5):1486. doi: 10.3390/s20051486 (PMC7085516; doi:10.3390/s20051486)

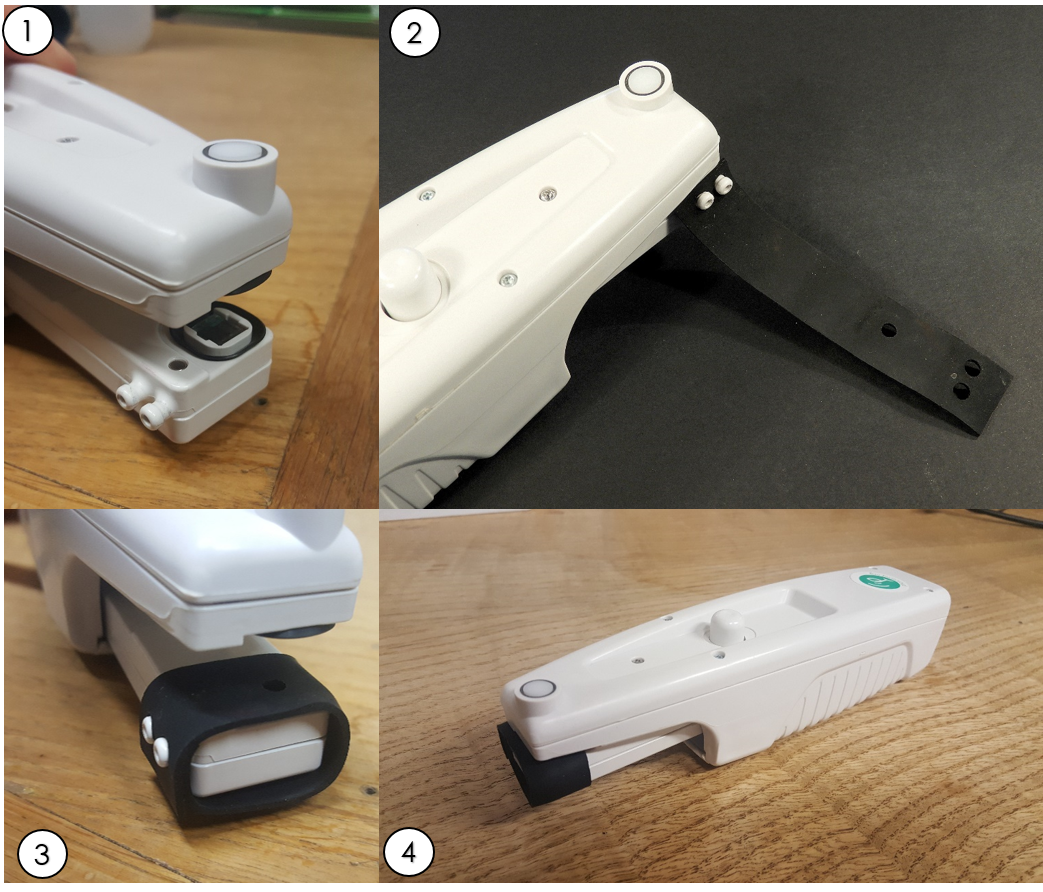

Supplement: Supplementary file 1 [file sensors-20-01486-s001.zip › FigureS1.png]
